# Tongue involvement in embouchure dystonia: new piloting results using real-time MRI of trumpet players

**DOI:** 10.1186/s40734-019-0080-3

**Published:** 2019-11-12

**Authors:** Soenke J. Hellwig, Peter W. Iltis, Arun A. Joseph, Dirk Voit, Jens Frahm, Erwin Schoonderwaldt, Eckart Altenmüller

**Affiliations:** 1 Hochschule für Musik, Theater und Medien, Hannover, Germany; 2 Gordon College, Wenham, MA USA; 3 Biomedizinische NMR, Max-Planck-Institut für biophysikalische Chemie, Göttingen, Germany

**Keywords:** Magnetic resonance imaging, Real-time MRI, Brass playing, Embouchure dystonia, Focal dystonia, Tongue movements, Movement disorder

## Abstract

**Background:**

The embouchure of trumpet players is of utmost importance for tone production and quality of playing. It requires skilled coordination of lips, facial muscles, tongue, oral cavity, larynx and breathing and has to be maintained by steady practice. In rare cases, embouchure dystonia (EmD), a highly task specific movement disorder, may cause deterioration of sound quality and reduced control of tongue and lip movements. In order to better understand the pathophysiology of this movement disorder, we use real-time MRI to analyse differences in tongue movements between healthy trumpet players and professional players with embouchure dystonia.

**Methods:**

Real-time MRI videos (with sound recording) were acquired at 55 frames per second, while 10 healthy subjects and 4 patients with EmD performed a defined set of exercises on an MRI-compatible trumpet inside a 3 Tesla MRI system. To allow for a comparison of tongue movements between players, temporal changes of MRI signal intensities were analysed along 7 standardized positions of the tongue using a customised MATLAB toolkit. Detailed results of movements within the oral cavity during performance of an ascending slurred 11-note harmonic series are presented.

**Results:**

Playing trumpet in the higher register requires a very precise and stable narrowing of the free oral cavity. For this purpose the anterior section of the tongue is used as a valve in order to speed up airflow in a controlled manner. Conversely, the posterior part of the tongue is much less involved in the regulation of air speed. The results further demonstrate that healthy trumpet players control movements of the tongue rather precisely and stable during a sustained tone, whereas trumpet players with EmD exhibit much higher variability in tongue movements.

**Conclusion:**

Control of the anterior tongue in trumpet playing emerges as a critical feature for regulating air speed and, ultimately, achieving a high-quality performance. In EmD the observation of less coordinated tongue movements suggests the presence of compensatory patterns in an attempt to regulate (or correct) pitch. Increased variability of the anterior tongue could be an objective sign of dystonia that has to be examined in further studies and extended to other brass instruments and may be also a potential target for therapy options.

## Background

Playing a brass instrument on a professional level demands complex coordination skills and precise spatiotemporal control of many different muscles and movements. Especially trumpet players face major challenges, because not only coordination, but also physical strength is necessary to build up sufficient air pressure for playing in the high register [1]. One of the most important aspects of tone production, and probably also the most vulnerable, is the embouchure. About 64% of professional trumpet players suffer from temporary or chronic embouchure problems during their career with up to 17% having to leave their job due to sickness [2]. The objective assessment of the underlying muscular problems of embouchure dystonia (EmD) is complicated by the fact that most critical movements happen inside the body and can only be examined by modern medical imaging techniques. Recent advances towards real-time magnetic resonance imaging (MRI) now offer a powerful new tool for visualizing movements of the tongue, septum, oral cavity and glottis at high spatial and temporal resolution [3, 4]. Moreover, real-time MRI is non-invasive and does not alter the sensory awareness while playing inside the MRI magnet [5].

In preceding studies healthy French horn players were compared to musicians suffering from EmD [6, 7]. EmD is a highly task-specific movement disorder with painless cramping or loss of voluntary motor control of highly trained movements. It has a prevalence of about 1% in the general population of all professional musicians [8]. In brass players, 96% of diagnosed focal dystonia manifests as EmD with cramping as a symptom in about 26% of subjects [2]. Often EmD begins in a certain register and eventually spreads out to neighbouring registers [9]. It can also first affect certain techniques such as tongued attacks and then progress to other techniques such as slurs. Typically, onset is in the fourth decade with progression to incapability of professional performance within 3 years [10]. Phenotypes which not only affect the orbicularis oris muscle seem to involve other oral activities [11] such as swallowing and chewing and have a bad prognosis in terms of maintenance of professional status.

In this study we focussed on tongue movements in healthy trumpet players and in those with diagnosed EmD. The restriction to trumpet players reflects the specific physical performance requirements in comparison to other brass instruments [12].

## Methods

### Subjects

Ten healthy trumpet players without any embouchure problems and four professional trumpet players with EmD diagnosed by an expert specialized in dystonia (author E.A.) participated in this study. Most healthy instrumentalists were students at the Music University in Hanover, Germany, where access is highly competitive and admission is limited to excellent players with more than 10.000 h of cumulative live practice time. The subjects’ characteristics are summarized in Table 1. No participant had any known neurological disorder or health problem (other than EmD), which could affect the testing. Prior to MRI, all subjects gave written informed consent in compliance with the regulations established by the local ethics committee of the Max-Planck-Institute and signed a consent form for publication, including consent to publish the real-time MRI-videos.

**Table 1:** Characteristics of healthy and diseased trumpet players

Subj.	Health status	Age / years	Gender	Professional Status	Months since diagnosis	Symptoms
1	H	23	M	Semi-prof.	–	
2	H	26	M	Student	–	
3	H	21	F	Student	–	
4	H	44	M	Professor	–	
5	H	25	M	Student	–	
6	H	24	M	Student	–	
7	H	18	M	Student	–	
8	H	26	M	Student	–	
9	H	23	M	Student	–	
10	H	19	F	Student	–	
11	D	51	M	Professional	20	Tone initiation problems
12	D	59	M	Professional	17	Tongue cramping
13	D	54	M	Professional	60	Lip cramping, sensory lip deficits
14	D	47	M	Professional	49	Breaking up of tones, problems with slurring

H = healthy; D = diseased; M = male, F = female

### Protocol

All subjects performed a defined set of exercises on a custom-made, MRI-compatible trumpet, which was built by Richard Seraphinoff, Bloomington, IN, USA. The instrument consists of a non-ferromagnetic handmade brass bell, two flexible plastic tubes with increasing diameter and a plastic Kelly’s mouthpiece (KELLY MOUTHPIECES, Fond du Lac, WI, USA). It was built to simulate a valve-less natural trumpet and is pitched in the key of C.

**Fig. 1:**
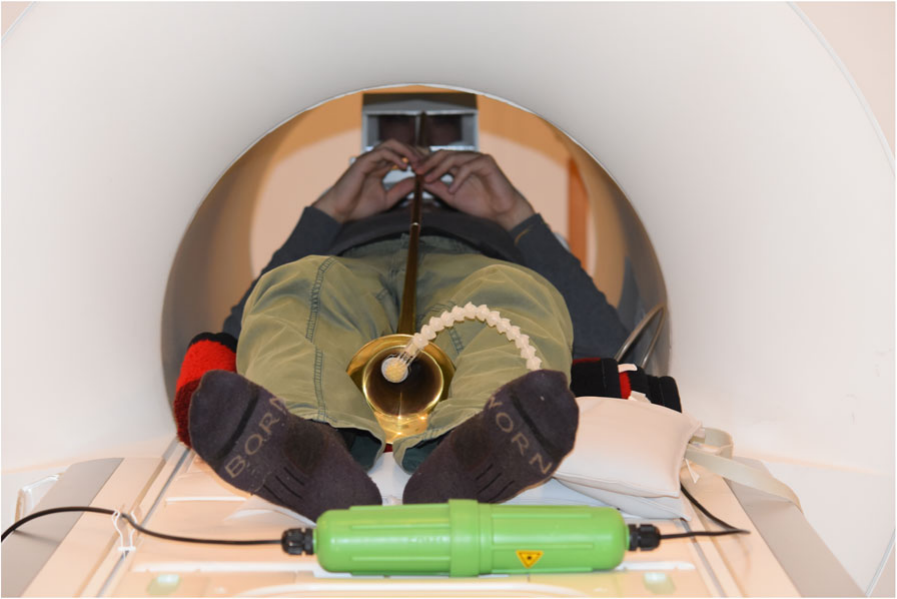
Setup for playing an MRI-compatible trumpet inside a 3 Tesla MRI system

The subjects played a set consisting of 19 different exercises, which were handed out to the participants one day before MRI. Each subject had about 5–10 min time to familiarize with the MRI-compatible trumpet before being positioned in the MRI magnet in order to play in a supine position. The bell of the trumpet was fixed at the end of the patient table with a microphone positioned inside the bell to get the best possible sound as demonstrated in Fig. 1. The other end of the tube with the plastic mouthpiece was inside the MRI head coil, where subjects held it with the fingers of one hand during playing, while serial MRI images (movies) were acquired. Despite the noise of the scanning procedure all subjects were able to hear themselves playing. Interaction with the investigators in the control room was ensured by a 2-way communication system. Prior to each exercise, an audio recording of the actual task was played to the participants.

**Fig. 2:**
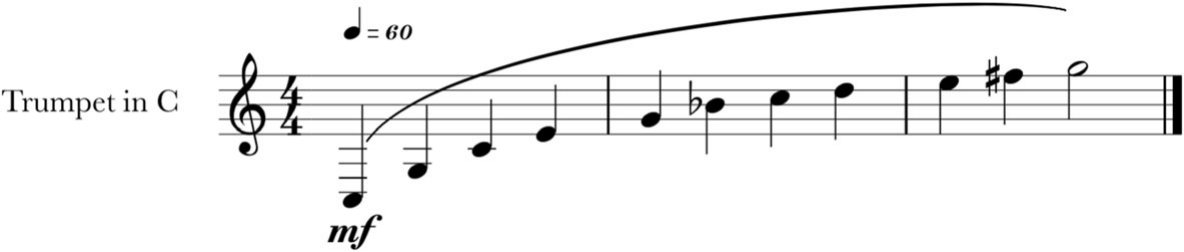
Ascending slurred 11-note harmonic series

In this study we focused on the analysis of a slurred 11-note ascending harmonic series in the low and middle range of the trumpet shown in Fig. 2, which is very comfortable to play. As it was the fifth task of the set of exercises, subjects were well acquainted with playing the instrument inside the MRI magnet. The exercise was chosen because it is common for trumpet players as part of their daily warm-up program.

### Real-time MRI

All MRI studies were performed at a field strength of 3 Tesla (Magnetom Prisma, Siemens Healthcare, Erlangen, Germany) using a 64-channel head coil. Real-time MRI was based on highly undersampled, radially encoded gradient-echo sequences (2.02 ms repetition time, 1.44 ms echo time, 5 degree flip angle, 9 spokes per image) in conjunction with serial image reconstruction by nonlinear inversion with temporal regularization [3]. The images had an in-plane resolution of 1.4 mm and a slice thickness of 8 mm covering a field-of-view of 192 × 192 mm^2^ with a resolution of 128 × 128 pixels. The majority of studies was performed at a temporal resolution of 18.18 ms (i.e., the image acquisition time) corresponding to 55 frames per second. The spatiotemporal accuracy of these methods was experimentally assessed [13] and allows detection of small object movements with velocities of up to 25 cm s^− 1^. Movies were obtained in a mid-sagittal orientation of the oral cavity to best cover tongue movements.

An MRI-compatible optical microphone (Dual Channel-FOMRI, Optoacoustics, Or Yehuda, Israel) was attached to the bell of the trumpet outside of the bore of the magnet, and sound recordings were subsequently synchronised to MRI movies as described elsewhere [4].

### Data analysis

**Fig. 3:**
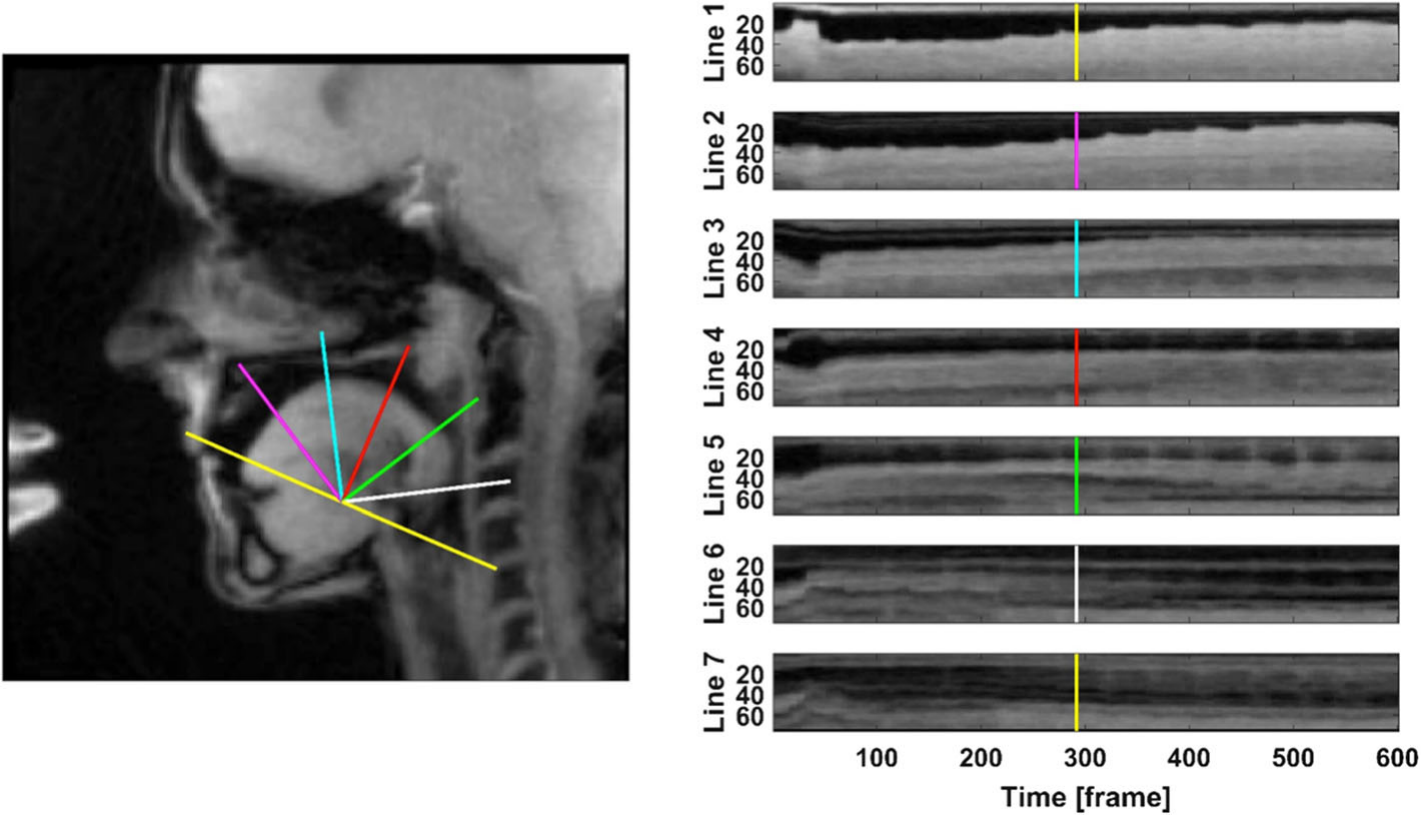
(Left) Sagittal image with definition of 7 profile lines and (right) resulting temporal intensity profiles (profile lines) for the ascending slurred 11-note harmonic series (vertical marker line positioned at the 5th note of the harmonic series)

For data analysis a customized real-time MRI toolbox for MATLAB (MATLAB R2015b, including ‘Image Processing Toolbox’, ‘Signal Processing Toolbox’ and ‘Statistics and Machine Learning Toolbox’) was employed as previously described [6, 7, 14–16]. The toolbox offers an individually positioned grid which covers the serial images for each subject. The assumed tip of the upper incisors and the inferior ventral edge of the fourth vertebra were used as anatomical landmarks. Based on the line drawn between two selected landmarks, the toolbox generates 7 lines of equal length, which differ by an angle of 30°. As demonstrated in Fig. 3, line 1 points to the tip of the upper incisors, line 4 to the uvula and line 7 to the inferior ventral edge of the fourth vertebra. A series of signal intensity profiles was generated for each line and serial image, which then allows a dynamic visualization of tongue movements along the respective direction. Temporal changes of these intensity profiles during playing are most pronounced at the edge of the tongue (proximal to the origin) and at the edge of the lip, hard palate, soft palate or spinal column (depending on the actual profile line) distal to the origin. Using the software, the location of these edges was calculated along each line for the entire exercise. For the purpose of this study, three profile lines were used for analysis: profile line 2 (PL2), PL5, and PL7 representing the anterior, middle and posterior oral cavity regions, respectively.

By defining the start and end frame of each note of the 11-note harmonic series with use of standard audio processing software (Audacity), we used the previously located positions of the two opposite edges inside the oral cavity to calculate a mean position of these edges along the three profile lines at each of the 11 tones. For a temporal resolution of 18.18 ms per frame, a tone of 1 s duration yields 55 values, which were used to compute mean values and their standard deviations. Using the length of each line from the origin to the distal edge to represent the entire oral cavity (OC), we calculated the percentage of that cavity comprised of the open space (% OCS) for each note.

**Fig. 4:**
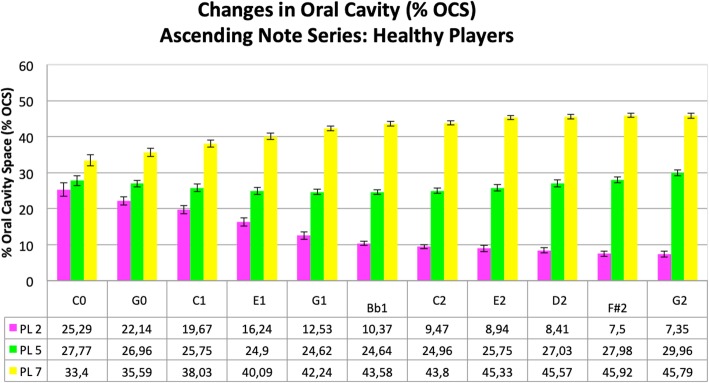
Changes in oral cavity (in % OCS and SD) during the ascending harmonic series in healthy trumpet players. Profile line 2 (pink), profile line 5 (green) and profile line 7 (yellow). Corresponding numeric values below

**Fig. 5:**
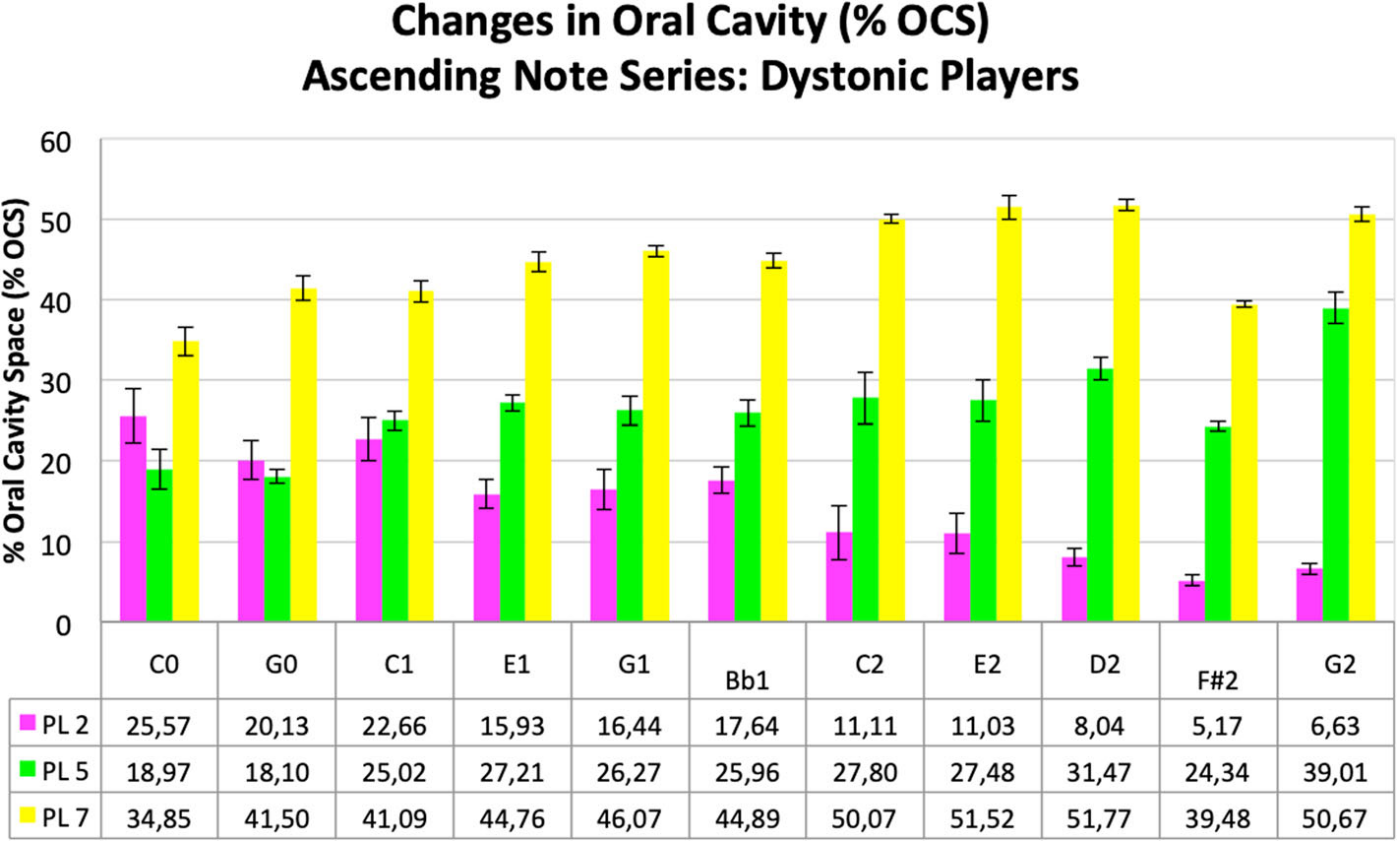
Changes in oral cavity (in % OCS and SD) during the ascending harmonic series in trumpet players with EmD. Profile line 2 (pink), profile line 5 (green) and profile line 7 (yellow). Corresponding numeric values below

In order to make all of the acquired data visible and to identify common features, the results for all healthy trumpet players were averaged across subjects for each of the three profile lines, and are presented in Fig. 4. The results of the dystonic trumpet players were averaged as well and are shown in Fig. 5.

**Fig. 6:**
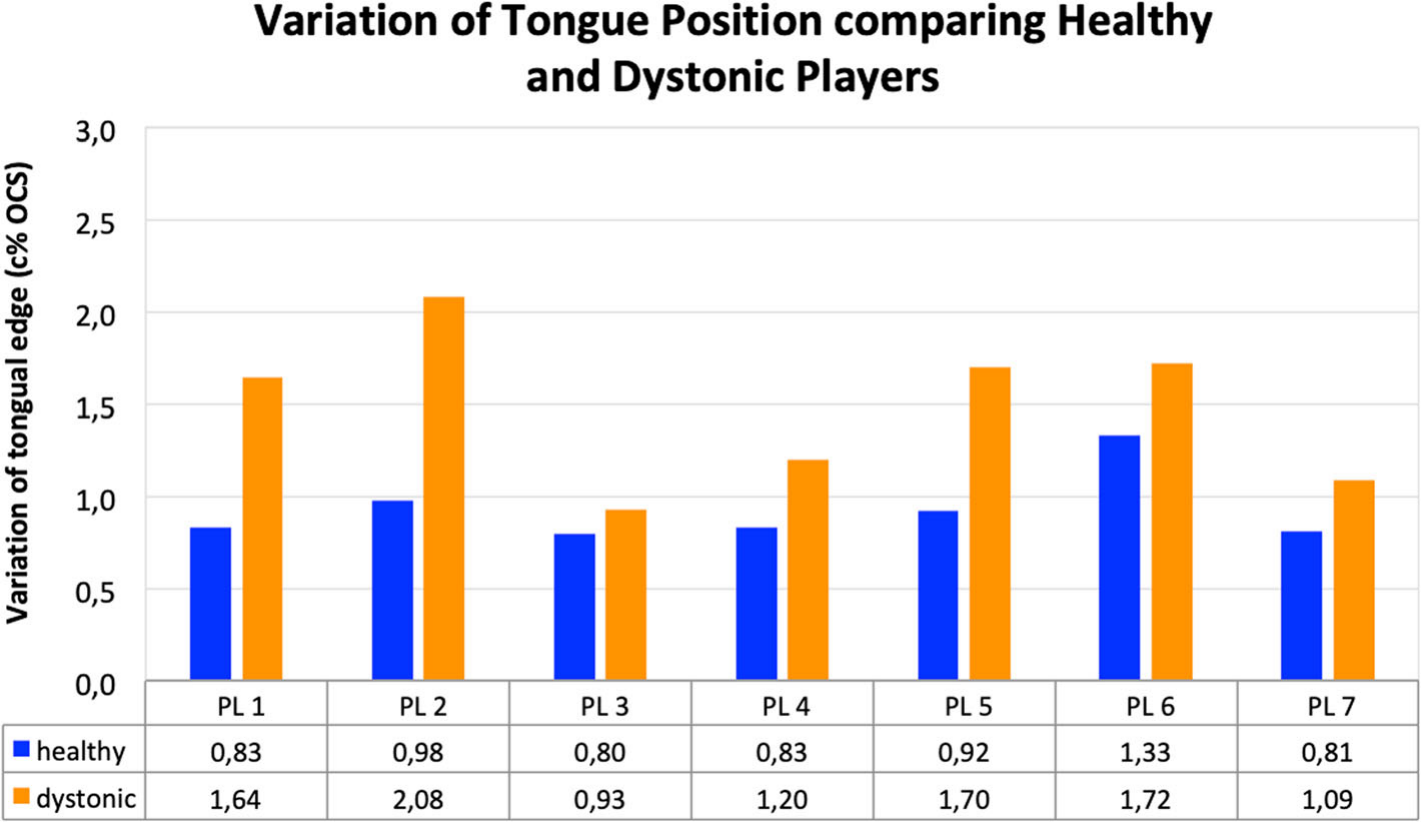
Variation of tongue position (c% OCS) for each profile line during the performed harmonic series for the healthy trumpet players (blue) and the trumpet players with EmD (orange). Corresponding numeric values below

To compare the stability of tongue position between groups, the standard deviations of the tongue edge during each note played along every profile line and for each performer were determined. These standard deviations were then set into relation with the corresponding OCS, so they show the %-change of the OCS for each note of the ascending harmonic series (c% OCS). The average c% OCS within both groups was then calculated along each profile line and is presented in Fig. 6.

## Results

### Healthy musicians

The results for the healthy subjects are presented in Fig. 4. One subject had to be excluded due to inadequate performance of the task. The data for the healthy trumpet players reveal several noteworthy aspects of tongue movements and oral cavity shape. First of all, when increasing pitch frequencies, there is a continuous decrease in % OCS, particularly in the anterior section of the oral cavity (profile line 2). A quantitative evaluation of the second profile line resulted in a reduction from 25,29% OCS (lowest tone) to 7,35% OCS (highest tone). Furthermore movements of the intermediate section represented by profile line 5 show a quite constant position of the tongue during the ascending harmonic series with 27,77% OCS in the lowest tone and 29,96% OCS in the highest. The pharyngeal section of the tongue (represented by profile line 7) demonstrates an inverted behaviour compared to the anterior section of the tongue such that the gap between the posterior tongue and pharyngeal structures increases rather than decreases for higher pitches. The corresponding % OCS values for profile line 7 increase from 33,4% to 45,79% with ascending notes (see also Additional file 1: Video 1).


Additional file 1: **Video 1.** Real-time MRI video of a healthy trumpet player performing a slurred 11-noste harmonic series.


### Patients with EmD

The results for the trumpet players with EmD are presented in Fig. 5. When looking at the anterior section of the tongue again represented by profile line 2, the % OCS also reduces with ascending the harmonic series from the lowest to the highest tone from 25,57% to 6,63% but not as continuously during the harmonic series as in the healthy trumpet players. The intermediate section of the tongue (see profile line 5) also behaves differently in the dystonic players, causing a gradual increase in % OCS from 18,97% to 39,01% during the ascending harmonic series rather than remaining constant. As with the healthy players, the pharyngeal section of the tongue moves anteriorly during the harmonic series, increasing the % OCS in this area from 34,85% on the lowest note to 50,67% on the highest tone. However, the consistency of this pattern was not as clear in the dystonic players (see also Additional file 2: Video 2).


Additional file 2: **Video 2.** Real-time MRI video of a trumpet player with embouchure dystonia performing a slurred 11-note harmonic series.


### Variation of the tongue position

In Fig. 6, the average c% OCS-values of the tongue edge position for each profile line of the healthy and dystonic trumpet players are presented. The maximum difference is illustrated in profile line 2 where the healthy trumpet player’s variation of the tongue position is 0,98 c% OCS and the variation of the trumpet players with EmD is 2,08 c% OCS. In profile lines 3 and 4 the difference is not as prominent. However, in profile line 5, the difference between the c% OCS-values of healthy and diseased trumpet players is again very strong. This difference is again lower in profile lines 6 and 7. Although the differences between the variations of the tongue position of healthy and diseased trumpet players vary between profile lines, the values representing the trumpet players with EmD are always higher compared to the healthy trumpet players.

## Discussion

This work presents for the first time high-resolution real-time MRI examinations of a group of healthy professional trumpet players and a small group of four professional trumpet players with EmD. Focus of the study was on the role of the tongue in controlling the size of the oral cavity, and on the variability of tongue position during each note in an ascending harmonic sequence. Although the sample size is limited, and the phenomenology of the four patients is somewhat variable and furthermore sound quality was not directly assessed, the study identified several mechanistic differences in tongue movement, which may help to explain the altered performance of players affected by EmD relative to that of healthy trumpet players.

First, healthy trumpet players decrease the distance between the surface of the tongue and the upper limit of the oral cavity (“close the gap”) when playing notes of increasing height. As described in Bernoulli’s law, voluntary narrowing of the free air channel accelerates the airflow to reach the speed for vibrating the lips at higher frequencies. A similar principle is shown in French horn players using real-time MRI [6]. Interestingly, and despite minor individual variations, this decrease of gap size in trumpet players seems to be restricted to the anterior section of the oral cavity (profile lines 1 to 3) and does not hold true for the intermediate and pharyngeal parts of the tongue.

In principle, trumpet players with EmD generated a similar pattern of tongue movements for the 11-note harmonic series, though with largely increased variability while sustaining each tone and a less consistent decrease of the %OCS as the healthy musicians. Similar to the group of healthy musicians, there was hardly any change in the intermediate part of the tongue. This finding again supports the notion that the airflow in trumpet players is mainly controlled by the anterior section of the tongue which seems to work as a valve. The slight increase of the gap size in the pharyngeal section of the oral cavity seen in both, EmD patients and healthy musicians may be the result of a high intra-oral pressure [1] and a passive stretching of the surrounding tissues by the amount of air inside the oral cavity.

When comparing healthy trumpet players and patients with EmD, two findings become obvious: (i) Healthy players are characterized by a very stable tongue position with well-defined movements of the anterior section when playing an ascending harmonic series. (ii) Musicians with EmD have difficulties in maintaining a stable tongue position during each note and apparently suffer from a lack of voluntary tongue control. This problem may indeed be a most characteristic symptom of dystonia suggesting a much more prominent role of the tongue in EmD than previously suggested. In terms of pathophysiology, two possible mechanisms have to be considered: 1.) variable tongue movements are compensation attempts to maintain stability of air-guidance in a dysfunctional “air-guidance-embouchure-system”, with primary loss of control in parts of the respiratory and oral tract, such as pharynx, facial muscles or even abdominal breathing muscles. 2.) lack of control of tongue movements is the primary cause, due to a lack of precision in spatiotemporal movement planning. In consequence, instability in control of air, leading to audible alteration in sound production, such as instable pitch, noise etc. may lead automatically via auditory-sensorimotor integration to compensatory facial and pharyngeal muscle activation. This may affect the lip aperture, and thus generate effects in more distant muscles, e.g. the buccinator muscle, which finally lead to a general breakdown of the sensorimotor program for control of sound production accompanied by subjective feelings of tension and cramping. Independently, whether the tongue instability is cause or consequence of primary dystonic movements, it is this holistic air-guidance-embouchure program that needs to be recovered by retraining or medical interventions. One future option of the real-time MRI methodology will be the use of visual feedback, which allows trumpet players to see the movements of their own tongue in real time while playing in the MRI magnet. This visual feedback might be a possibility to reinforce self-awareness and to accelerate the retraining process in the event of dysfunctional movements.

## Conclusion

The present study underlines the importance of proper tongue movements in professional trumpet players both in healthy subjects and patients suffering from EmD. In particular, the regulation of pitch appears to be mainly controlled by the anterior portion of the tongue, which requires very precise and stable movements, especially when playing in the higher register. For trumpet players with EmD, the regain of control of the precision in the movements of the anterior part of the tongue may be an adequate way of improving the playing ability. Therefore further therapy-oriented research is required as more efficient treatment strategies are urgently needed [11]. Based on the present findings, we suggest a retraining-therapy that should focus on the anterior section of the tongue in order to regain precision, stability and control. Further real-time MRI studies of musicians with and without EmD should compare tongue movements in different brass instruments in order to gain further insights into mechanistic alterations and possible therapeutic options.

## Data Availability

The real-time MRI data of the study is stored in the Max-Planck-Institut für biophysikalische Chemie, Göttingen, Germany. Please contact author Jens Frahm for data requests. The further processed data of the study are stored in the Institute of Music Physiology and Musicians Medicine, at the University of Music, Drama, and Media Hannover. Please contact. Dr. Eckart Altenmüller.

## Abbreviations

EmD: Embouchure Dystonia
MRI: Magnetic Resonance Imaging
OC: Oral Cavity
OCS: Oral Cavity Space
